# Anthropomorphic Measurements That Include Central Fat Distribution Are More Closely Related with Key Risk Factors than BMI in CKD Stage 3

**DOI:** 10.1371/journal.pone.0034699

**Published:** 2012-04-12

**Authors:** Philip D. Evans, Natasha J. McIntyre, Richard J. Fluck, Christopher W. McIntyre, Maarten W. Taal

**Affiliations:** 1 Department of Renal Medicine, Royal Derby Hospital, Derby, United Kingdom; 2 Department of Vascular Medicine, The University of Nottingham, Derby Campus, Derby, United Kingdom; University of Sao Paulo Medical School, Brazil

## Abstract

**Background:**

Body Mass Index (BMI) as a marker of obesity is an established risk factor for chronic kidney disease (CKD) and cardiovascular disease (CVD). However, BMI can overestimate obesity. Anthropomorphic measurements that include central fat deposition are emerging as a more important risk factor. We studied BMI, waist circumference (WC), waist-to-height ratio (WHtR), waist-to-hip ratio (WHR) and conicity index (CI) in a cohort of patients with CKD stage 3 and compared the associations with other known risk factors for CKD progression and CVD.

**Methods:**

1740 patients with CKD stage 3 were recruited from primary care for the Renal Risk in Derby study. Each participant underwent clinical assessment, including anthropomorphic measurements and pulse wave velocity (PWV), as well as urine and serum biochemistry tests.

**Results:**

The mean age of the cohort was 72.9±9 years with 60% females. The mean eGFR was 52.5±10.4 ml/min/1.73 m^2^ and 16.9% of the cohort had diabetes. With the cohort divided into normal and increased risk of morbidity and mortality using each anthropomorphic measurement, those measurements that included increased central fat distribution were significantly associated with more risk factors for CKD progression and CVD than increased BMI. Univariable analysis demonstrated central fat distribution was correlated with more risk factors than BMI. Subgroup analyses using recognised BMI cut-offs to define obesity and quartiles of WHR and CI demonstrated that increasing central fat distribution was significantly associated with more CKD and CVD risk factors than increasing BMI.

**Conclusion:**

Anthropomorphic measurements that include a measure of central fat deposition are related to more key risk factors in CKD stage 3 patients than BMI. Central fat deposition may be of greater importance as a risk factor in CKD than BMI and reliance on BMI alone may therefore underestimate the associated risk.

## Introduction

Overweight and obesity, defined by body mass index (BMI; kg/m^2^), are associated with increased risk of hypertension [1], diabetes [2], malignancy [3] and mortality [4]. In addition, an association between obesity and chronic kidney disease (CKD) has been identified over the past 10 years. Several population-based, observational studies showed obesity, defined by BMI, as an independent risk factor in the development of CKD [5], [6] and end stage kidney disease (ESKD) [7]. In other studies overweight, obesity and increased central fat distribution (as defined by waist-to-hip ratio, WHR) have been associated with reduced estimated glomerular filtration rate (eGFR) and microalbuminuria [8]. In African Americans with hypertensive nephrosclerosis, BMI was shown to be independently associated with urinary protein and albumin excretion [9]. Recently, increasing waist circumference (WC) categories were associated with an increased mortality risk in a population with CKD stages1-4 [10]. The effect of obesity in haemodialysis patients is more controversial with a number of studies reporting a reduction in the relative risk of mortality with increasing BMI [11] but the inclusion of a measure of central fat deposition associates with an increased mortality risk [12], [13]. Current UK CKD management guidelines recommend routine BMI measurement but do not recommend assessment of central fat distribution [14].

There is mounting evidence to suggest that BMI may not be the ideal measure of obesity, especially when used to assess disease risk. BMI is indiscriminate; including fat mass and muscle mass in its measurement [15]. Therefore, a person with increased muscle mass, but normal fat mass, could have a raised BMI and be wrongly defined as overweight or obese. BMI does not account for the differing distributions of body fat between individuals or populations [15]. Increased abdominal fat deposition is associated with insulin resistance [15], is a stronger risk factor for morbidity and mortality than peripheral fat deposition [16] and varies greatly within a narrow BMI range [15].

A number of other methods for assessing obesity related morbidity that include measures of abdominal fat deposition have been proposed for assessing obesity related health risk. WC and WHR are the most commonly used methods and are associated with obesity-related morbidity and mortality [17]. Waist-to-height ratio (WHtR) correlates well with CT assessment of intra-abdominal fat [18] and one study found it to be more strongly associated with cardiovascular disease (CVD) risk than WHR, WC or BMI [19]. Conicity Index (CI) is a measure that includes weight, height and WC; it demonstrates good association with WHR [20] and at increasing levels has been associated with increased mortality risk in a haemodialysis population [13].

To examine which method for assessing obesity-related health risk may be the most appropriate in patients with CKD we studied the relationship between a number of different anthropomorphic measurements and established risk factors for CKD progression and CVD in a community-based cohort with CKD stage 3.

## Methods

### Subjects and recruitment

A detailed description of the methods has been published previously and is summarised here with emphasis on the anthropomorphic variables studied [21], [22]. The Renal Risk In Derby (RRID) study was conducted from a single Nephrology Department. Subjects were directly recruited from community medical care practices. Study visits were conducted at participating community medical centres by the researchers. Eligible participants were adult, met the KDOQI criteria for CKD stage 3 (eGFR 30–59 mL/min/1.73 m^2^ on 2 or more occasions at least 3 months apart prior to recruitment), able to give informed consent and attend their GP surgery for assessments by the researchers. People who had previously had a solid organ transplant or were terminally ill (expected survival <1 year) were excluded.

### Data collection

We combined screening and baseline visits due to the logistical challenges associated with conducting study visits in multiple primary care centres and the large proportion of elderly participants. Each participant was sent a medical and dietary questionnaire and 3 urine specimen bottles. They were requested not to eat cooked meat for at least 12 hours prior to the assessment [23].

At the assessment anthropomorphic measurements were taken (height, weight, WC and hip circumference, HC). Blood specimens were taken and with urine specimens were submitted for biochemical analysis. Blood pressure (BP) and pulse wave velocity (PWV) were also measured. Diabetes was defined by having a previous clinical diagnosis in line with WHO criteria [24].

### Anthropomorphic Measurements

Weight and height measurements were taken at assessment. WC was measured as per WHO guidelines; at the mid-point between the lower border of the rib cage and the iliac crest. HC was measured at the widest point of the hips and the maximal protrusion of the gluteal muscles [17].

BMI was computed as weight (in kilograms) divided by the square of the height (in metres). WHR ratio was computed as the ratio of WC to HC. WHtR was computed as the ratio of WC to height. CI was calculated using the following formula:
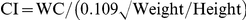

[20] Overweight was defined as a BMI ≥25–29.9 kg/m^2^ and obesity defined as a BMI ≥30 kg/m^2^. Central fat distribution was defined as a WHR of ≥0.9 for men or ≥0.85 for women; a WC of >94 cm for Europid males, >90 cm for Asian/Chinese/Japanese males and >80 cm for all females; a WHtR of >0.5 for males and females [17].

### Blood pressure

BP was measured after a minimum of five minutes rest in the sitting position, using an oscillometric device, recommended by the British Hypertension Society. The same device was used for all readings. BP was calculated as the mean of three readings that differed by <10% [25].

### Pulse wave velocity

PWV was measured as a marker of arterial stiffness, a critical determinant of cardiovascular outcomes in CKD [26], [27]. Measurements were performed using a Vicorder™ device (Skidmore Medical Ltd, Bristol, UK) between carotid and femoral arteries.

### Albuminuria

Albuminuria was assessed by measuring the urine albumin to creatinine ratio (UACR) on three consecutive early morning urine specimens collected prior to the clinic visit and stored in a refrigerator [23].

### Estimation of glomerular filtration rate

Biochemical assessments were performed by autoanalyser in a single laboratory. The creatinine assay has been standardised against an isotope dilution mass spectrometry (IDMS) method. The modified 4-variable MDRD equation was used to estimate GFR.

### Ethics

The study was approved by the Nottingham Research Ethics Committee 1 and abides by the principles of the Declaration of Helsinki. All participants provided written consent. The study was included on the National Institute for Health Research (NIHR) Clinical Research Portfolio (NIHR Study ID:6632) and was independently audited by QED Clinical Services in November 2009.

### Statistical Analysis

Variables are reported as the mean and standard deviation (SD) if normally distributed or the median and inter-quartile range (IQR) if not. A t-test was used to compare groups where variables were normally distributed and a Mann-Whitney test used if not. Univariable linear regression analysis was used to evaluate independent associations between anthropomorphic variables (independent variables) and risk factors for CKD progression or CVD (eGFR, Log UACR, PWV and serum uric acid used as dependent variables). SPSS version 16.0 was used for analysis and p<0.05 was considered statistically significant.

## Results

Overall 22% of the approximately 8,280 potential participants invited agreed to be included in the study. A total of 1822 subjects were enrolled between July 2008 and March 2010, 81 were excluded because they did not meet the inclusion criteria despite being on a CKD register at their GP practice; 1 further patient was excluded due to incomplete anthropomorphic data. Thus a total of 1740 participants were included in this analysis.

Baseline data are shown in Table 1. The mean weight was 78.2±15.5 kg and mean BMI 29±5.1 kg/m^2^. Forty two percent of the cohort were classified overweight (BMI ≥25–29.9 kg/m^2^) and 37% classified obese (BMI ≥30 kg/m^2^). Seventy three percent of the cohort had increased central fat distribution as defined by WHR (≥0.9 for men or ≥0.85 for women) [17].

**Table 1:** Baseline Data.

	Cohort (n = 1740)
**Age (years)**	72.9±9
**Female**	1052(60.4)
**SBP(mmHg)**	134±18
**Diabetes**	294(16.9)
**eGFR (ml/min/1.73 m^2^)**	52.5±10.4
**UACR (mg/mmol)**	0.33[0–1.5]
**Uric Acid (mmol/L)**	384.2±91.2
**PWV (m/sec)**	9.9±2.0
**Weight (Kg)**	78.2±15.5
**BMI (kg/m^2^)**	29±5.1
**WC (cm)**	97.6±12.8
**WHR**	0.91±0.09
**WHtR**	0.6±0.07
**CI**	1.3±0.09

Data are mean±SD, median [IQR] or number (%).

SBP = systolic blood pressure; eGFR = estimated glomerular filtration rate; UACR = urine albumin to creatinine ratio; PWV = pulsewave velocity; BMI = body mass index; WC = waist circumference; WHR = waist-to-hip ratio; WHtR = waist-to-height ratio; CI = conicity index.

The cohort was divided into overweight or obese and non-overweight defined by BMI, or normal risk and increased risk of metabolic complications, defined by WC, WHtR and WHR. CI was excluded from this analysis as currently no agreed cut-off for increased risk exists in the literature. Increased BMI and WC were associated with only a minority of risk factors for CKD progression and CVD but WHtR and WHR were each associated with multiple factors (Table 2).

**Table 2:** Comparison between cohort divided into normal and raised BMI; normal and raised WC; normal and raised WHR; normal and raised WHtR.

	BMI		WC		WHtR		WHR	
	Normal (n = 353)	Overweight+Obese (n = 1387)	Normal (n = 257)	Increased Risk (n = 1483)	Normal (n = 144)	Increased Risk (n = 1596)	Normal (n = 471)	Increased Risk (n = 1269)
**Age (years)**	73.6±10	72.7±8.7	72.8±10.2	72.9±8.8	70.4±11.4	73.1±8.8*	72.1±9.9	73.2±8.7#
**eGFR (mL/min /1.73 m^2^)**	52.6±10.8	52.5±10.3	52.8±10.8	52.4±10.3	54.2±11.3	52.3±10.3#	53.9±10.4	52.0±10.3*
**SBP (mmHg)**	132.7±20.4	134.3±17.8	132.5±20	134.2±18	129.6±20.1	134.4±18.1#	132.7±18.5	134.5±18.2
**UACR (mg/mmol)**	0.3[0–1.8]	0.33[0–1.44]	0.3[0–1.38]	0.37[0–1.5]	0.15[0–0.85]	0.37[0–1.57]#	0.2[0–0.91]	0.43[0.06–0.13]*
**Uric acid (mmol/L)**	358.1±86.	390.8±91.*	354.4±86	389.3±91*	325.1±80	389.5±90*	349.5±85	397±90*
**PWV (m/sec)**	10.0±2.0	9.9±2.0	9.8±2.1	9.9±2.0	9.7±2.1	9.9±2.0	9.8±2.1	9.9±2.0

* p≤0.001;

# p<0.05.

Data are mean±SD, median [IQR] or (number).

eGFR = estimated glomerular filtration rate; SBP = systolic blood pressure; UACR = urine albumin to creatinine ratio; PWV = pulsewave velocity; BMI = body mass index; WC = waist circumference; WHtR = waist-to-height ratio; WHR = waist-to-hip ratio.

Univariable analyses were performed between continuous anthropomorphic measurements and risk factors associated with CKD progression and CVD. Increasing BMI, WC and WHtR were significantly correlated with a minority of risk factors whereas WHR and CI were significantly correlated with multiple risk factors (Table 3).

**Table 3:** Univariable analysis between continuous anthropomorphic measures and established risk factors for CKD progression or cardiovascular events.

	BMI		WC		WHtR		WHR		CI	
Dependent Variable	Beta	Std.Error	Beta	Std.Error	Beta	Std.Error	Beta	Std.Error	Beta	Std.Error
**eGFR (mL/min/1.73 m^2^)**	−0.06	0.05	−0.09*	0.02	−13.70*	3.31	−20.37*	2.77	−16.89*	2.63
**SBP(mmHg)**	0.03	0.09	0.06	0.03	5.42	5.87	10.52#	4.96	9.61#	4.69
**Log UACR**	0.004	0.007	0.017*	0.003	2.015*	0.495	3.860*	0.411	3.100*	0.391
**Uric acid (mmol/L)**	3.48*	0.42	2.20*	0.16	295.74*	28.37	317.20*	23.58	257.92*	22.63
**PWV (m/sec)**	−0.053*	0.01	0.001	0.004	−0.776	0.652	2.776*	0.544	2.962*	0.513

* p<0.001;

# p<0.05.

All Coefficients reported are unstandardised.

eGFR = estimated glomerular filtration rate; SBP = systolic blood pressure; UACR = urine albumin to creatinine ratio; PWV = pulsewave velocity; BMI = body mass index; WC = waist circumference; WHtR = waist-to-height ratio; WHR = waist-to-hip ratio; CI = conicity index.

The cohort was then divided into subgroups using recognised BMI cutoffs (≤24.9; 25–29.9; 30–39.9; ≥40), WHR quartiles and CI quartiles. Increasing BMI was associated with a significant increase in serum uric acid concentration whereas PWV decreased with increasing BMI (Table 4). In contrast WHR and CI were comparably associated with a significant change in multiple risk factors (Tables 5 and 6).

**Table 4:** Comparison between BMI subgroups and risk factors for CKD progression or cardiovascular events.

	BMI (kg/m^2^)				
	<25 (n = 353)	25–29.9 (n = 738)	30–39.9 (n = 589)	40+ (n = 60)	p
**Age(yrs)**	73.59±10.0	73.79±8.6	71.71±8.7	68.8±8.5	<0.001
**SBP(mmHg)**	132.7±20.4	134.7±17.6	133.8±18.2	133.1±15.6	0.352
**eGFR (mL/min/1.73 m^2^)**	52.6±10.8	52.9±10.4	52.1±10.1	51.3±10.3	0.459
**UACR(mg/mmol)**	0.3[0–1.8]	0.37[0–1.5]	0.33[0–1.4]	0.17[0–1.13]	0.102
**Uric acid (mmol/L)**	358.05±86.2	379.75±89.1	402.49±90.5	412.82±108.3	<0.001
**PWV(m/sec)**	9.99±2.0	10.17±2.0	9.59±1.9	8.87±1.7	<0.001

Data are mean±SD or median [IQR].

SBP = systolic blood pressure; eGFR = estimated glomerular filtration rate; UACR = urine albumin to creatinine ratio; PWV = pulsewave velocity; BMI = body mass index.

**Table 5:** Comparison between WHR quartiles and risk factors for CKD progression or cardiovascular events.

	WHR Quartiles				
	1^st^ (n = 376)	2^nd^ (n = 476)	3^rd^ (n = 419)	4^th^ (n = 469)	p
**Age(yrs)**	71.43±10.1	72.64±8.8	73.35±9.1	73.81±8.2	0.001
**SBP(mmHg)**	132.2±18.9	133.4±18.6	135.7±17.9	134.5±17.8	0.049
**eGFR (mL/min/1.73 m^2^)**	54.03±10.3	53.95±10.7	52.51±10.3	49.80±9.7	<0.001
**UACR(mg/mmol)**	0.17[0–0.87]	0.23[0–0.9]	0.42[0–1.7]	0.77[0.07–3.4]	<0.001
**Uric acid(mmol/L)**	346.09±84.0	369.39±87.7	398.21±87.5	416.99±89.4	<0.001
**PWV(m/sec)**	9.68±2.1	9.63±2.0	10.10±2.0	10.16±2.0	<0.001

Data are mean±SD or median [IQR].

SBP = systolic blood pressure; eGFR = estimated glomerular filtration rate; UACR = urine albumin to creatinine ratio; PWV = pulsewave velocity; WHR = waist-to-hip ratio.

**Table 6:** Comparison between CI quartiles and risk factors for CKD progression or cardiovascular events.

	CI Quartiles				
	1^st^ (n = 426)	2^nd^ (n = 427)	3^rd^ (n = 445)	4^th^ (n = 442)	p
**Age(yrs)**	70.75±10.0	72.15±8.7	73.56±8.5	74.89±8.3	<0.001
**SBP(mmHg)**	132.0±19.1	134.4±17.2	135.2±17.9	134.3±19.0	0.058
**eGFR (mL/min/1.73 m^2^)**	54.0±10.5	53.4±10.3	52.0±10.3	50.6±10.2	<0.001
**UACR(mg/mmol)**	0.2[0–0.79]	0.23[0–1.03]	0.42[0–1.8]	0.77[0.07–3.4]	<0.001
**Uric acid(mmol/L)**	348.21±84.7	380.44±89.0	401.06±86.4	405.22±93.5	<0.001
**PWV(m/sec)**	9.57±2.1	9.67±1.8	10.05±2.0	10.28±2.0	<0.001

Data are mean±SD or median [IQR].

SBP = systolic blood pressure; eGFR = estimated glomerular filtration rate; UACR = urine albumin to creatinine ratio; PWV = pulsewave velocity; CI = conicity index.

The analysis was repeated with diabetic subjects excluded and all associations observed in the whole cohort were reproduced (data not shown). Further subgroup analyses were performed with the cohort divided into age <75 yrs; age ≥75years and CKD stage 3a (eGFR: 60-46 mL/min/1.73 m^2^) ; CKD stage 3b (eGFR: 45-30 mL/min/1.73 m^2^).

Patients aged ≥75 yrs had a significantly higher mean uric acid (p<0.001), PWV(p<0.001), SBP(p<0.001), WHR (p = 0.007) and CI(p<0.001) and a significantly lower mean eGFR(p<0.001) and BMI (p<0.001) compared with those aged <75 yrs. Comparisons between normal and raised anthropomorphic measures, univariable analyses and comparisons between BMI subgroups and WHR, CI quartiles were similar in both age groups to those of the full cohort. The CKD3a results were similar to those of the full cohort; unfortunately the CKD3b group was statistically under-powered (n = 410) and produced no significant results (data not shown).

## Discussion

This study shows that anthropomorphic measurements that include central fat distribution are associated with more established risk factors for CKD progression and CVD than BMI.

On the basis of age, eGFR and albuminuria the RRID study cohort is broadly representative of patients with CKD followed up in primary care, in the UK [28]. A greater percentage of the cohort was overweight and obese (defined by BMI) than would be expected from general UK population data (79% with CKD stage 3 compared to 66% of men and 57% of women in the general population) and this trend continued when comparing proportions of obesity alone (37% with CKD stage 3 compared with 22% men and 24% of women from the general population) [29].

When examining the relationship between anthropomorphic cut-offs for increased risk and other risk factors, a raised WHR was significantly associated with more risk factors than the other measures. The association with increasing age is supported by previous findings of increasing all-cause mortality in a cohort aged ≥70 years with increasing WHR; a similar relationship was not found with BMI [30]. This highlights another limitation of BMI which tends to decrease in the elderly (as demonstrated with the association of decreasing mean age as BMI groups increased in our cohort – Table 4) as the decrease in BMI is a reflection of the loss of skeletal muscle mass experienced with aging as opposed to loss of fat mass [30].

A raised WHR was also significantly associated with a decreased eGFR, increased UACR and serum uric acid. The association with decreased eGFR agrees with previous findings reporting a relationship between increasing WHR and diminishing glomerular filtration [8] as well as incident CKD and mortality [31]. This relationship with eGFR is important given that decreasing eGFR is associated with an increased risk of CV and all-cause mortality [32]. The association of increased WHR with the increased UACR is significant given that UACR has been found to be a stronger risk factor for progression of CKD than low baseline eGFR [33] and increased UACR increases the risk of CV and all cause mortality [32]. Mean serum uric acid levels, identified in previous studies as an independent CV risk marker in subjects with [34] and without CKD [35], [36], [37], were also increased in the raised WHR group.

Univariable analyses demonstrated WHR and CI correlating with more risk factors than any other anthropomorphic measure. WC and WHtR were associated with similar, but fewer, risk factors to WHR and CI and all include measures of central fat deposition. Unexpectedly BMI was negatively correlated with PWV, a surrogate marker of arterial stiffness. Increased arterial stiffness is emerging as a major risk factor for CV disease [38], [39] and has been identified as an independent risk factor for CV death in the general population [40] as well as in subjects with CKD [26], [41]. This finding is therefore contrary to what we would expect from the literature [26], [42], [43]. Our observation may be explained by the fact that BMI decreased with increasing age in our study population and age is a major determinant of increasing PWV [44]. In contrast, WHR did not decrease with increasing age, suggesting that it is a better marker of obesity in an elderly population.

Dividing the cohort into subgroups using recognised BMI cut-offs, WHR quartiles and CI quartiles demonstrated a significant difference between the subgroups in more risk factors for CKD progression and CV disease with WHR and CI quartiles than with BMI subgroups.

Our data suggest an advantage to having a measurement of central fat distribution when assessing risk of CKD progression and CVD in a cohort with CKD stage 3 and we offer the following explanation for this. A number of factors have been implicated in the pathogenesis of obesity-related kidney disease, such as: hyperfiltration, excess fatty acid accumulation in tissue (lipotoxicity), increased insulin resistance and alterations in circulating adipokine levels [45]. Adipose tissue is a large endocrine organ secreting a number of different adipokines. Obese subjects have a different adipokine profile compared to lean subjects with obese subjects having increased levels of pro-inflammatory adipokines (PIAs). Obesity is therefore associated with low-grade inflammation [46] that has been proposed as a causative link between obesity and its complications [47]. The mechanisms that provoke increased secretion of PIAs await further elucidation. Hypoxic conditions in animals and in-vitro in white adipose tissue are associated with increased PIA secretion [46] and peri-operative measurement of oxygen levels in abdominal adipose tissue showed a decreased oxygen tension in obese subjects, when compared to non-obese [48]. Therefore, increased tissue hypoxia observed with increasing central fat distribution may be a driving factor underlying obesity-related morbidity. It is only by measuring central fat distribution that this effect can be taken into account.

Interestingly, as all anthropomorphic measures of obesity increased, serum uric acid levels also rose significantly within the cohort. An increased serum uric is associated with increased CV risk [49]. It is raised in CKD and the metabolic syndrome [49]. Whether it is actually a risk factor for CKD progression and the metabolic syndrome is yet to be proven, and the subject of ongoing research [49].

Strengths of this study include confirmation of CKD stage 3 by two eGFR values (often lacking in epidemiological studies), serum creatinine measurements performed after a 12 hour meat fast and samples assayed in a single laboratory using an assay standardised to an IDMS method as well as urine chemistry repeated on three consecutive early morning specimens to minimise the effect of daily variation in low levels of albuminuria. We have therefore established a robust baseline dataset that will allow detailed analysis of the predictive value of differing anthropomorphic measurements as potential risk factors when outcome data become available.

Several limitations of this study need to be acknowledged. Despite rigorous application of the entry criteria, some subjects had an eGFR≥60 mL/min/1.73 m^2^ at their first clinical visit. Follow up will yield important data on the natural history and associated risks of this subgroup. For ethical reasons we were obliged to rely on eligible patients volunteering to participate and this strategy may have introduced an element of bias, since those more concerned about their health would be more likely to participate. As discussed, however, the RRID cohort is broadly representative of patients followed up in Primary Care with CKD stage 3, though it may not be representative of all such populations. The cohort includes only a minority of subjects of African or Asian origin as the population in Derbyshire is predominantly Caucasian. Several studies have reported a high prevalence of CKD and obesity in African and Asian populations associated with a higher risk of CKD progression and our observations may therefore not be directly applicable to these ethnic groups.

### Conclusion

Anthropomorphic measurements that include a measure of central fat deposition are related to more key risk factors in CKD stage 3 patients than BMI. Central fat deposition may be of greater importance as a risk factor in CKD than BMI and reliance on BMI alone may therefore underestimate the associated risk. This may be particularly important in elderly patients, in whom BMI tends to decrease with age.

Long term follow up of this cohort will allow assessment of the value of these anthropomorphic measurements to predict outcomes and to identify high risk groups for possible intervention. Further studies are required to investigate the efficacy of interventions to reduce obesity related risks.
